# RETRACTION: Circular RNA circFBXO11 Modulates Hepatocellular Carcinoma Progress and Oxaliplatin Resistance Through miR‐605/FOXO3/ABCB1 Axis

**DOI:** 10.1111/jcmm.70355

**Published:** 2025-01-11

## Abstract

**RETRACTION**: J. Li, X. Qin, R. Wu, L. Wan, L. Zhang, and R. Liu, “Circular RNA circFBXO11 Modulates Hepatocellular Carcinoma Progress and Oxaliplatin Resistance Through miR‐605/FOXO3/ABCB1 Axis,” *Journal of Cellular and Molecular Medicine* 24, no. 9 (2020): 5152–5161, https://doi.org/10.1111/jcmm.15162.

The above article, published online on 28 March 2020 in Wiley Online Library (wileyonlinelibrary.com), has been retracted by agreement between the journal Editor‐in‐Chief, Stefan Constantinescu, The Foundation for Cellular and Molecular Medicine and John Wiley and Sons Ltd. The retraction has been agreed upon following an investigation into concerns raised by a third party, which revealed implausible similarities between Figure 2G NC of this study and another article published earlier in the same year by a different group of authors in a different scientific context, suggesting that the images were likely obtained from the same flow‐cytometry data set. The explanation and the partial raw data shared by the authors were deemed insufficient to address these concerns. Thus, the editors have lost confidence in the presented data and consider the conclusions of this manuscript substantially compromised.

**RETRACTION**: J. Li, X. Qin, R. Wu, L. Wan, L. Zhang, and R. Liu, “Circular RNA circFBXO11 Modulates Hepatocellular Carcinoma Progress and Oxaliplatin Resistance Through miR‐605/FOXO3/ABCB1 Axis,” *Journal of Cellular and Molecular Medicine* 24, no. 9 (2020): 5152–5161, https://doi.org/10.1111/jcmm.15162.

The above article, published online on 28 March 2020 in Wiley Online Library (wileyonlinelibrary.com), has been retracted by agreement between the journal Editor‐in‐Chief, Stefan Constantinescu, The Foundation for Cellular and Molecular Medicine and John Wiley and Sons Ltd. The retraction has been agreed upon following an investigation into concerns raised by a third party, which revealed implausible similarities between Figure 2G NC of this study and another article published earlier in the same year by a different group of authors in a different scientific context, suggesting that the images were likely obtained from the same flow‐cytometry data set. The explanation and the partial raw data shared by the authors were deemed insufficient to address these concerns. Thus, the editors have lost confidence in the presented data and consider the conclusions of this manuscript substantially compromised.

